# Molecular factors associated with lung cancer in people living with HIV

**DOI:** 10.1016/j.bjid.2026.105791

**Published:** 2026-02-19

**Authors:** Ivonne Denisse Bautista-Rojas, Jesus Figueroa-Navarrete, Diana Laura Reyes-Hernandez, Evelyn Rivera-Toledo

**Affiliations:** Universidad Nacional Autónoma de México (UNAM), Facultad de Medicina, Departamento de Microbiología y Parasitología, Mexico City, Mexico

**Keywords:** HIV, Lung cancer, Non-AIDS-defining cancers, Oncogenic HIV proteins

## Abstract

Lung cancer is the most common Non-AIDS-Defining Cancer (NADC) and a leading cause of cancer-related death in People Living With HIV (PLWH). Despite antiretroviral therapy, PLWH are at higher risk of developing cancer compared to the general population. This increased susceptibility reflects a combination of immunosuppression, chronic inflammation, smoking, and direct oncogenic effects of HIV proteins. Tat, gp120, and Nef modulate cell cycle control, apoptosis, epithelial-mesenchymal transition, angiogenesis, and immune evasion. Persistent HIV reservoirs in lung tissue (mainly effector memory CD4⁺ T-cells and alveolar macrophages) sustain local immune dysregulation. Extracellular vesicles carrying viral proteins or nucleic acids activate oncogenic pathways, while HIV integration disrupts tumor suppressor genes such as PTEN and induces epigenetic silencing of regulators like P16^INK4a^. These alterations, together with oxidative stress, promote a pro-tumorigenic microenvironment. A deeper understanding of these mechanisms may enable early biomarker identification and the design of targeted preventive and therapeutic strategies for lung cancer in PLWH.

## Introduction

Human Immunodeficiency Virus (HIV), the etiology of the Acquired Human Immunodeficiency Syndrome (AIDS), caused 1.3 million of new infections and 630,000 AIDS-related deaths in 2024, to record 40.8 million (37.0 million–45.6 million) people living with HIV (PLWH).^1^

Currently, 77 % of the total infected population have access to the Antiretroviral Therapy (ART). Consequently, the incidence of HIV-related opportunistic infections has been significantly reduced.^1^ Nevertheless, high-risk groups among PLWH still exhibit high rates of oropharyngeal and esophageal candidiasis,^2^ bacterial pneumonia,^3^ herpes zoster^4^ and tuberculosis.^5^

Moreover, HIV is associated with an increased risk of cancer development, which is a leading cause of HIV-associated death.^6^ Cancers directly associated with T-cell immunodeficiency are classified as AIDS-Defining Cancers (ADCs), including Kaposi Sarcoma (KS), Non-Hodgkin Lymphoma (NHL), and cervical cancer. Conversely, non-AIDS-Defining Cancers (NADCs) can occur in both HIV-positive and HIV-negative individuals, although their incidence is higher in people living with HIV.^7^

Forty different types of NADCs have been recognized, with approximately half of them being associated with oncogenic viruses such as Epstein-Barr, human papillomavirus and hepatitis B and C viruses. These cancers with infectious etiology such as anal, vulvar, vaginal, Hodgkin lymphoma and liver malignancies exhibit high incidence. NADCs without infectious etiology include mesothelial and soft tissue tumors, multiple myeloma and lung cancer. Notably, people living with HIV show higher progression and mortality rates for these malignancies compared to the HIV-negative population.^8^

In this review, we focus on the molecular mechanisms associated with the development of lung cancer as a NADC of clinical relevance. This multifactorial malignancy requires continued research to elucidate its mechanisms of pathogenesis and identify risk factors to implement targeted therapies and preventive strategies.

## Literature search strategy

This article is a descriptive review. A comprehensive literature search was conducted in the databases PubMed and Google Scholar. The search focused on key terms related to HIV infection and cancer pathogenesis, including HIV, cancer, oncogenic HIV proteins, lung cancer, non-AIDS-defining cancers, pathogenesis, etiology, and molecular mechanisms. The search was primarily limited to articles published within the last 10-years and written in English. Review articles, epidemiological studies, and treatment-focused studies were excluded during the initial search to prioritize original research addressing molecular and pathogenic mechanisms. Articles were selected based on their relevance to the scope of the review.

## HIV pathogenicity

HIV is an enveloped lentivirus that primarily targets CD4+ cells expressing chemokine receptors, particularly CCR5 and CXCR4, which serve as coreceptors during viral entry. The envelope proteins gp120 and gp41 mediate receptor recognition and fusion of the viral envelope with the plasma membrane, allowing entry of the nucleocapsid.^9^ Once internalized, the viral RNA is reverse-transcribed into double-stranded DNA while the nucleocapsid migrates to the nucleus. The viral DNA is subsequently imported through nuclear pores and integrated into the host genome. During the late stage of the replication cycle, viral genes are transcribed, the genome is replicated, and newly assembled virions are released from the infected cell.^10^

Virions using CCR5 as a coreceptor (R5 viruses) initially target memory CD4+ *T*-cells, which are enriched in mucosal tissues. This early phase of infection results in a massive CD4+ *T*-cell destruction by direct viral cytopathic effects, cytotoxic T-lymphocyte-mediated killing, and bystander apoptosis induced by Env-mediated signaling. Acute HIV infection is associated with viral loads ranging from 10^4^ to 10^7^ RNA copies/mL^11^ and leads to the establishment of viral reservoirs, which consist of latently infected cells harboring integrated replication-competent proviruses.

The HIV reservoir is primarily established in resting CD4+ *T*-cells, which become infected during the transition from an activated to a quiescent state, as well as in myeloid cells residing in different tissues. Accordingly, major viral reservoirs have been identified in the bone marrow,^12^ lymph nodes, the gut-associated lymphoid system, spleen, thymus, and central nervous system.^13^^,^^14^

Even though ART is effective in reducing plasma viremia to undetectable levels and has improved the quality of life and expectancy for HIV-infected individuals, it does not eliminate the virus from the latently infected reservoir, which carries inducible proviruses capable of reactivation.^15^ Sampling of different CD4+ *T*-cell subsets from lymph nodes, Gut Associated Lymphoid Tissue (GALT) and peripheral blood mononuclear cells has revealed that these are active anatomical compartments functioning as sanctuaries for HIV persistence.^16^ Furthermore, HIV DNA has been detected in specialized macrophage populations such as Kupffer cells, alveolar macrophages and microglia, as well as in mucosal cells of the penile urethra and vagina, contributing to the complexity and heterogeneity of the HIV reservoir.^17^

In addition to replication-competent proviruses, some cells harbor defective proviral genomes with internal deletions. Although these defective proviruses are incompetent to produce infectious viruses, they can express viral proteins that contribute to chronic inflammation and T-cell exhaustion. In consequence, there exist increased risk to develop HIV-associated comorbidities such as cardiovascular disease, neurodegeneration and cancer.^18^, ^19^, ^20^

## HIV proteins with oncogenic effects

**Fig. 1:**
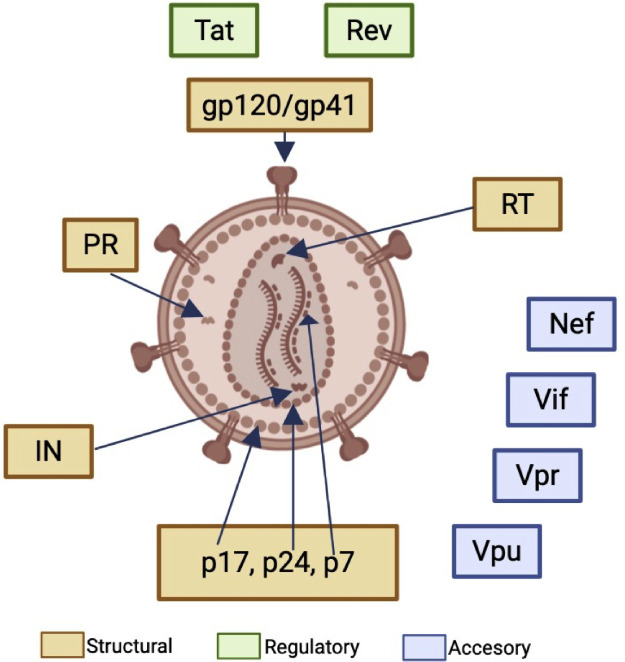
HIV-1 structural, regulatory and accessory proteins. The *Gag* gene encodes the structural proteins matrix (p17), capsid (p24) and nucleoprotein (p7), in addition to p6 (not shown) that mediates viral release. The *Pol* gene encodes the enzymes Reverse Transcriptase (RT), Integrase (IN) and Protease (PR). The *Env* gene encodes the envelope glycoproteins gp120/gp41. Created with BioRender.

The HIV genome encodes the *Gag, Pol* and *Env* genes which express structural, regulatory and accessory proteins (Fig. 1). Among the regulatory proteins, Tat (trans-activator of transcription) and Rev (regulator of RNA splicing) control viral gene transcription, while accessory proteins such as Nef (Negative regulating factor), Vif (Viral infectivity factor), Vpr (Virus protein *r*) and Vpu (Virus protein unique) regulate replication, viral budding, immune evasion and pathogenesis.^21^ Interestingly, the envelope protein gp120, Nef and Tat have been implicated in HIV-associated oncogenesis.

Tat is a small regulatory protein that increases RNA polymerase II activity during viral genome transcription and downregulates MHC-I expression, contributing to immune evasion and viral persistence. It is secreted at high concentrations in the supernatants of HIV-infected lymphocytes, as well as in serum and cerebrospinal fluid from patients, even under viral control with antiretroviral therapy.^22^

Tat has also been linked to modulation of the Tat-Interacting Protein 30 (TIP30), a known tumor suppressor involved in regulating apoptosis, proliferation, metastasis, angiogenesis, DNA repair, and tumor metabolism in various cancers, including Hepatocellular Carcinoma (HCC), colorectal carcinoma, glioma, breast cancer, laryngeal squamous cell carcinoma, esophageal carcinoma, lung cancer, among others.^23^

In hepatocellular carcinoma, loss of TIP30 promotes Epithelial-Mesenchymal Transition (EMT). During the EMT the transcription factor Snail is overexpressed; it has been associated with invasive breast cancer cells since it recruits a repressor complex to inhibit *E-cadherin* transcription.^24^ Nuclear translocation of Snail is facilitated by its interaction with importin-β2. Tat interacts with importin-β2 increasing nuclear localization of Snail leading to EMT, enhanced cell motility and poor prognosis by metastasis.^25^^,^^26^

The envelope glycoproteins gp120 and gp41 are membrane-associated proteins, although gp120 can also be released in a soluble form stimulating neighboring cells and altering signaling pathways related to immune response, cell viability and cell cycle regulation.^27^^,^^28^ Particularly, gp120 induces IL-6 production and expansion of myeloid derived suppressor cells with a phenotype CD11b^+^, CD33^+^, HLA-DR^-/low^, and pSTAT3^hi^, which are associated with expansion of regulatory T-cells and immunosuppression.^29^ Additionally, gp120 induces apoptosis and disrupt the cell cycle in epithelial, lymphoid and neuronal cells by upregulating cyclin dependent kinase 1 and proapoptotic effectors such as Bax, Fas/FasL, caspase 3 and caspase 8.^30^^,^^31^

Soluble gp120 has also profibrotic activity in human hepatic stellate cells through interaction with CXCR4 and subsequent ERK1/2 phosphorylation, triggering collagen I expression. This process possibly accelerate liver fibrosis in HIV positive individuals, especially those coinfected with hepatitis C virus.^32^

In epithelial cells, both gp120 and Tat upregulate markers of EMT, such as N-cadherin and vimentin, along with downregulation of E-cadherin and cytokeratin.^33^ Immunohistochemical analysis of cervical cancer tissues from HIV-positive women coinfected with Human Papillomavirus (HPV) show diminished expression of E-cadherin and cytokeratin, whereas vimentin is increased with respect to tissues from HIV-negative patients, suggesting that gp120 and Tat induce EMT and facilitate the progression of HPV-associated cervical lesions.^33^

**Fig. 2:**
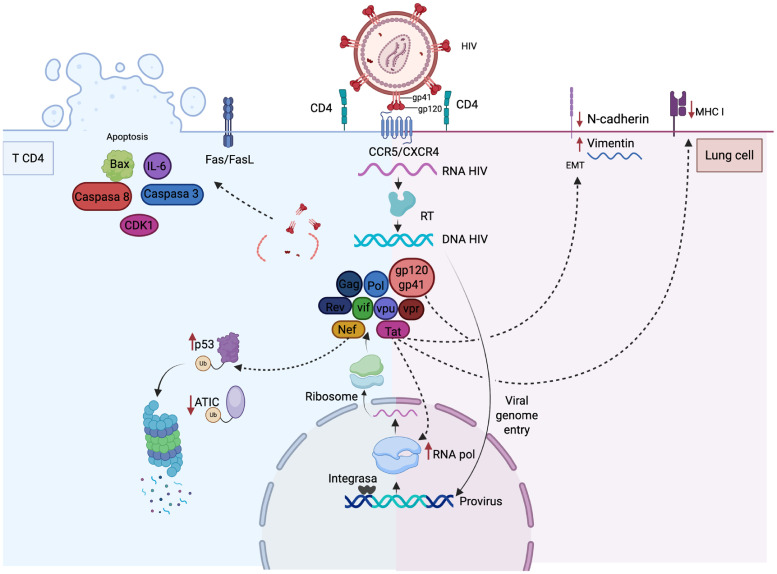
HIV entry is mediated by the interaction of the viral envelope proteins gp120/gp41 with the host cell molecules CD4 (primary receptor) and CCR5 or CXCR4 (main coreceptors). After the Reverse Transcriptase (RT) enzyme synthesize the viral DNA and it is imported to the nucleus, the integrase inserts the viral DNA into the host-cell chromosomes. The transcription, replication and translation of the viral genome are mediated by hijacked cellular components, which allows for the synthesis of all necessary components to assemble a new viral progeny. HIV replication is optimized by processes such as immune evasion and cell survival. The viral protein Tat not only increases viral transcription but also reduces the expression of MHC-I in epithelial and lymphoid cells. Both Tat and gp120 induce expression of vimentin while E-cadherin is downregulated, an event associated with the EMT. In addition, soluble gp120 induces the synthesis of IL-6, which indirectly promotes the expansion of Treg lymphocytes, upregulates CDK1 (to alter the cell cycle), and induces the expression of proapoptotic molecules. Nef modulates the stability of both viral and cellular proteins by ubiquitination (e.g., inducing p53 degradation via the proteasome) and increases the expression of the enzyme ATIC, which upregulates the proto-oncogene Myc in lung cancer. Created with BioRender.

On the other hand, the regulatory protein Nef is expressed early after infection and manipulates host cell processes to increase viral replication and persistence. Nef modulates T-cell activation by internalization and lysosomal degradation of CD4 and CD28. Promotes indirectly viral infectivity of CD4^+^ *T*-cells through production of CCL2 and CCL4 in macrophages, which results in chemotaxis and activation of T-lymphocytes, which become permissive to HIV (Fig. 2).^34^ Nef also contributes to immune evasion avoiding antigen presentation by interfering of the trafficking of HLA-I, reducing recognition of infected cells by cytotoxic lymphocytes.^35^ Its role in modulating stability of viral and cellular proteins that participate in virus replication via the ubiquitin proteasome system has been described by proteomic analysis.^36^ Actually, Nef interacts with the ubiquitin E3 ligase E6AP to induce p53 ubiquitination and proteasomal degradation.^37^ P53 is a transcriptional regulator in response to DNA damage induced by ultraviolet light, gamma radiation and carcinogens. Loss of p53 function impairs cell cycle control and apoptosis, allowing genetically damaged cells to survive and accumulate oncogenic mutations.^38^

In addition, Nef increases the expression of the enzyme 5-aminoimidazole-4-carboxamide ribonucleotide formyltransferase/IMP cyclohydrolase (ATIC), which participates in the purine biosynthetic pathway.^36^ ATIC is upregulated in myeloma and HCC. Also, this enzyme is overexpressed in advanced stages of lung adenocarcinoma and promotes cell growth and migration through upregulation of the proto-oncogen Myc.^39^

Lung cancer, as all types of cancers, is multifactorial and particularly, in people living with HIV, immunosuppression, chronic inflammation, smoking and the oncogenic HIV proteins are interconnected triggering factors. In the following sections we summarize epidemiology, evidence of HIV persistence in lungs and molecular mechanisms associated with lung cancer in PLWH.

## Lung cancer in PLWH

Lung cancer is the most common NADC and the leading cause of cancer-related deaths in PLWH.^40^^,^^41^ Histologically, it is classified in two main types: Non-Small Cell Lung Cancer (NSCLC) and small cell lung cancer. NSCLC is a heterogeneous group of epithelial malignancies that account for 86 %–94 % of lung cancers; the main subtypes are adenocarcinoma, squamous cell carcinoma and large cell carcinoma.^42^ Adenocarcinoma is the most frequent subtype (30 %–50 %).

The incidence of lung cancer in patients with HIV represents a notable disparity compared to the general population. Epidemiological studies indicate that PLWH have 1.5–3-fold increased risk of developing lung cancer, compared with the HIV-negative population.^42^^,^^43^

A study using data from the multicenter CoRIS Spanish cohort (2004‒2021) reported 176 deaths associated with non-AIDS-defining tumors, of which, 36.4 % were related to lung cancer (mortality rate of 0.57, 95 % CI 0.45, 0.73). Lung cancer was associated with a 2-fold increase in mortality in PLWH. The risk of mortality was also higher in association with factors such as coinfection with hepatitis B and C viruses, heterosexual HIV transmission, use of injected drugs, active smoking, and CD4 counts < 500 µL.^44^

Another study that analyzed data from the HIV/AIDS Cancer Match Study (USA) from 2001 to 2016 estimated a significant decrease in the incidence of lung cancer among individuals with HIV: incidence rates were 124.4 per 100,000 person-years during 2001 to 2003, compared to 58.3 per 100,000 person-years in the period from 2013 to 2016. However, the incidence rates remained 48 % higher in HIV^+^ individuals than in the general population.^42^ Such decrease could be associated with increased access to ART; however, immune recovery is incomplete in a proportion of patients and may represent a persistent risk factor for lung cancer. In particular, a low CD4/CD8 ratio, a low nadir CD4+ *T*-cell count and residual or intermittent viremia despite ART have been associated with higher incidence of this malignancy in PLWH. Moreover, aging and pulmonary infections related to chronic inflammation (e.g., tuberculosis) may also contribute to cancer risk. Of relevance, lung cancer diagnosis in PLWH has been associated with decreased median survival compared with general population (12.4 vs. 22.8-months).^41^, ^42^, ^43^

## HIV persistence in lung tissue

In addition to the gut-associated lymphoid tissue, other long-term HIV reservoirs have been identified.^45^ Particularly, HIV provirus has been quantified in Bronchioalveolar Lavages (BAL) and Peripheral Blood Leucocytes (PBLs) from patients living with HIV that developed respiratory symptoms. Results revealed higher levels of proviral DNA in BAL derived cells (2971 HIV DNA copies/10^6^ cells), compared to PBLs (391 HIV DNA copies/10^6^ lung cells). Interestingly, viral genome can be detected even in patients receiving nucleoside reverse-transcriptase inhibitors, although with reduced levels (up to 6-fold decrease).^46^

HIV DNA has also been quantified in BAL from patients without respiratory symptoms, under ART and with a minimum of 3-years of viral suppression. In this case, BAL derived cells showed higher HIV DNA copies than PBMCs (13-fold); particularly, effector memory CD4^+^ *T*-cells were the principal HIV reservoir. Interestingly, the pulmonary CD4^+^ *T*-cell population showed an enrichment of CD57^+^ cells that are associated with immuno-senescence, whereas T-regulatory Foxp3^+^ cells were also increased. Thirty percent of the patients showed HIV RNA in BAL derived cells, indicating active viral genome transcription.^47^

Notably, Nef^+^ cells (CD4^+^, CD8^+^ and alveolar macrophages) have been evidenced in BAL from patients with undetectable levels of p24 and genomic RNA. Therefore, persistent expression of HIV regulatory proteins in lung tissue and other anatomical sites contribute to tissue damage, immunosuppression and cancer development.^48^

## Mechanisms associated with lung cancer in HIV

### Virus derived components and extracellular vesicles

Studies in human respiratory tract derived cell lines indicate that Nef induces cell proliferation and migration, as well as apoptosis resistance when it is transfected in A549 cells and non-cancerous BEAS-2B cells. Also, Nef increases expression of angiogenesis and tissue remodeling factors such as Nuclear Factor of Activated T-cells-1 (NFAT-1), Matrix Metalloproteinase-9 (MMP-9) and Vascular Endothelial Growth Factor (VEGF-A) whereas it reduces p53 protein levels (Table 1).^49^ Processing of micro-RNA (miRNAs) is also altered in BEAS-2B cells transfected with Nef, since this viral protein significantly reduces the level of expression of the enzymes DICER and Ago2. Consequently, miRNAs associated with lung cancer are downregulated, indicating this viral protein impairs miRNA biogenesis, contributing to tumor development and cancer progression.^49^

**Table 1:** Function of proteins differentially altered in lung cancer tissues.

**Molecule**	**Role in cancer**	**Up or down regulation in lung cancer**	**Reference**
NFAT-1	Controls cell cycle, migration, invasion and apoptosis. Induce angiogenesis and matrix metalloproteinases expression.	Up	^57^
MMP-9	Metalloproteinase-9, promotes tumor invasion and metastasis by tissue remodeling and angiogenesis.	Up	^49^
VEGF-A	Vascular Endothelial Growth Factor-A participates in angiogenesis, cell proliferation, migration and survival. Associated with immunosuppression in the tumor microenvironment.	Up	^49^
EMAP-II	Endothelial monocyte-activating polypeptide-II. Antiangiogenic cytokine that inhibits tumor growth and metastasis by inducing apoptosis.	Up	^48^
FOS	Proto-oncogene. Regulates cell proliferation, differentiation, stress response and apoptosis. Its overexpression is associated with metastasis.	Up	^75^
DEFB103	Beta-defensin. Regulates inflammatory response by modulating cytokines and chemokines. Involved in cell proliferation.	Up	^52^
c-Myc	Oncogene that regulates cell proliferation, metabolism and evasion of the host immune response, promoting cancer progression.	Up	^39^
SIX1	Sine Oculis Homeobox. Transcription factor involved in organ development during embryogenesis and in disorders such as cancer. Reactivates developmental programs.	Up	^76^
PROM1	Prominin-1. Membrane glycoprotein. Marker of cancer stem cells; it enhances tumorigenesis, metastasis, treatment resistance, and activation of pathways such as Wnt/β-catenin, PI3K-Akt, as well as VEGF-A/IL-8 production.	Up	^54^
TFAP2A	Oncogenic factor. Promotes tumor proliferation and angiogenesis. Also inhibits apoptosis. Member of the AP-2 transcription factors family.	Up	^54^
TOX3	Thymocyte selection-associated HMG BOX. Promotes proliferation, migration, invasion, resistance to apoptosis, and maintenance of the stem-like phenotype.	Up	^77^
SOX9	Transcription factor that regulates embryonic development, tissue differentiation, and maintenance of cancer stem cells. It promotes proliferation, invasion, resistance to apoptosis, and EMT.	Up	^78^
ADH1B	Catalyzes the oxidation of ethanol to acetaldehyde. It acts as an acetaldehyde detoxifier and prevents persistent oxidative stress.	Down	^54^
INMT	Indolethylamine N-methyltransferase. Tumor suppressor by limiting cell proliferation, migration and invasion.	Down	^54^
SYNPO2	Synaptopodin-2 directs cytoskeletal organization, limits proliferation, migration, invasion and EMT. Participates in autophagy	Down	^53^
P16^INK4a^	Inhibits the cell cycle and induces cellular senescence by blocking CDK4/6, preventing RB phosphorylation. Induces cell cycle arrests avoiding G1/S transition.	Down	^79^
P53	Tumor suppressor. Regulates the cell cycle, induces apoptosis, and maintains genomic stability.	Down	^38^
DICER	Endoribonuclease. Promotes post-transcriptional gene silencing by processing pre-miRNA to mature single-stranded forms.	Down	^49^
Ago2	Argonaute 2, participates in biogenesis and function of miRNAs and siRNAs.	Down	^80^
PTEN	Tumor suppressor. Controls chromatin condensation, centromere stability, accurate chromosome segregation and DNA replication.	Down	^56^

Interestingly, Nef has been detected in Bronchioalveolar Lavages (BAL) of HIV patients under ART, both into cells and Extracellular Vesicles (EVs). Nef^+^-EVs isolated from HEK 293T cells transfected with Nef induced expression and release of TNF-α, IL-6, MCP-1, CXCL10 and RANTES in primary human alveolar macrophages, contributing to chronic inflammation and increasing the risk for lung cancer. Also, Nef induced upregulation of the cytokine Endothelial Monocyte-Activating Polypeptide-II (EMAP-II), which is involved in oxidative stress and apoptosis, as well as in the pathogenesis of emphysema.^48^ Worth to mention that emphysema is a predisposing factor for lung cancer,^50^ as long-term inflammation involves pulmonary tissue damage and remodeling.^51^

On the other hand, it has been identified EVs with Gag and gp160 as cargo proteins, as well as the HIV Transactivation Response (TAR) RNA element that regulates viral transcription. EVs isolated from HIV-infected T-cells in vitro induced proliferation and migration of human head and neck squamous carcinoma cell lines. Such effect can be reproduced with EVs derived from plasma of HIV-infected individuals under ART, with respect to EVs from healthy individuals. Notably, the HIV-TAR RNA cargo was the effector element involved in expression of the proto-oncogenes FOS, DEFB103 and c-Myc through phosphorylation of ERK1/2 in an EGFR/TLR3-dependent manner, to induce expression of the proto-oncogenes.^52^

Lung cancer tissues derived from HIV+ patients have been studied to determine their transcriptional profile. A study with lung biopsies from lung adenocarcinomas, squamous cell carcinomas and small cell lung cancer indicated the most importantly upregulated genes were SIX1, PROM1, TFAP2A, TOX3 and SOX9, whereas ADH1B, INMT and SYNPO2 were significantly downregulated, in comparison with paired adjacent non-tumor tissues. Of such altered genes, SYNPO2 is a potential tumor suppressor in diverse types of cancers, whereas SIX1 has been associated with tumor development and progression. Also, SIX1 is a potential biomarker of clinical prognosis of patients with non-small cell lung cancer.^53^^,^^54^

### Effect of HIV integration into the host DNA and epigenetics

The HIV genome preferentially integrates into transcriptionally active genes. Proviral integration in genes related to cell growth has been observed in approximately 12.7 % of PBMCs, increasing to 16.0 % in proliferating cells. Gene ontology analysis indicated that unique HIV integrations were enriched in genes from cell proliferation pathways such as MAPK1, BACH2, C2CD3, CREBBP, FBX11, DNMT1, among others.^55^

A study in a patient with diagnosis of small cell lung carcinoma showed 174 unique HIV integration sites in lung tumor tissue, of which, 30 % were identified in clonally expanded cells. Viral DNA was principally integrated in the Phosphatase and Tensin Homolog (PTEN) gene that encodes an enzyme with phosphatase activity. Phosphatidylinositol-3,4,5-trisphosphate (PIP3) is a second messenger that triggers the PI3K/AKT pathway to promote cell survival, proliferation and differentiation. Active PTEN dephosphorylates the PIP3 to 4,5-bisphosphate acting as a tumor suppressor.^56^ Histopathological analysis showed that tissue regions expressing p24 showed loss of expression of PTEN protein,^57^ suggesting that PTEN downregulation contributes to lung cancer development and progression.

Notably, PTEN and other genes involved in cell cycle and DNA repair have been found hypermethylated in non-small cell lung cancer, particularly at promoter regions in CpG islands.^58^

Another gene silenced in early stages of non-small cell lung cancers by hypermethylation is the tumor suppressor p16^INK4a^,^59^ which regulates cell cycle progression by inhibiting the S phase.^60^ Interestingly, acute HIV infection of Hut 78 cells increases expression of the DNA methylase DNMT1 along with hypermethylation of the p16^INK4a^ promoter which is associated with significantly reduced gene transcription.^61^

Thus, epigenetics plays a significant role in lung cancer development, and patterns of methylation of specific genes in the Wnt signaling pathway can be used as prognostic biomarkers.^62^

### Altered immune response and oxidative stress

During HIV infection, T-cells are persistently stimulated and activated by viral antigens. This condition eventually triggers T-cell exhaustion associated with impaired cytokine production, upregulation of inhibitory receptors such as PD-1, CTLA-4 and TIM-3, as well as reduced proliferative potential. Functional T-cell damage leads to poor elimination of pathogens and cancer cells.^63^

On the other hand, a chronic state of oxidative stress has been described in PLWH.^64^ Reactive Oxygen and Nitrogen Species (ROS and RNS) are produced during lung inflammation, mainly by activated alveolar macrophages, neutrophils and epithelial cells.^65^ Oxidative stress is produced by an imbalance between oxidants and antioxidant effectors. The antioxidant activity is mediated by enzymes such as superoxide dismutase, catalase, glutathione peroxidase and thioredoxin.^66^ ROS alter the activity of transcription factors such as STAT-1, MAPKs and NF-kB to trigger inflammatory signals. Persistent exposure to ROS and RNS promotes macrophage senescence.^67^

Interestingly, primary alveolar macrophages from PLWH without respiratory symptoms release significantly increased levels of the superoxide anion, whereas their bronchioalveolar lavages display reduced levels of the antioxidant glutathione, in comparison to HIV negative individuals.^68^

A transgenic rat model expressing gp120 and Tat, but unable to assemble viral particles showed reduced levels of glutathione in lungs compared to wild type rats. In addition, pulmonary epithelium displayed increased paracellular permeability associated with reduced levels of the adhesion molecules zonula occludens-1 and occludin in the alveolar epithelium; such effect was related to the expression of the viral proteins.^69^ Also, mice expressing Tat in the lungs showed cellular infiltration and oxidative stress associated with higher levels of nitrotyrosine, manganese superoxide dismutase, and NF-kB (p65), supporting the role of Tat to induce the oxidative environment.^70^

Therefore, the oxidative stress mediated by HIV-proteins impairs the alveolar epithelial barrier by decreasing antioxidant effectors and altering expression of intercellular adhesion proteins. However, free radicals also produce genomic instability and trigger signaling pathways related to oncogene expression (e.g. Jun and Fos).^71^ Of interest, overexpression of Jun-D has been directly associated with lung cancer.^72^

Finally, smoking is a major risk factor for lung cancer in the general population and increases the risk up to five-fold in HIV^+^ patients.^73^ Potent carcinogens and immunosuppressive molecules derived from the combustion of tobacco such as isoprene, benzene, benzo[a]pyrene, carbon monoxide, hydrogen cyanide, nitric oxide, ROS, among others, alter lung function and contribute to systemic inflammation enhancing susceptibility to infections and development of malignancies.^74^

## Concluding

**Fig. 3:**
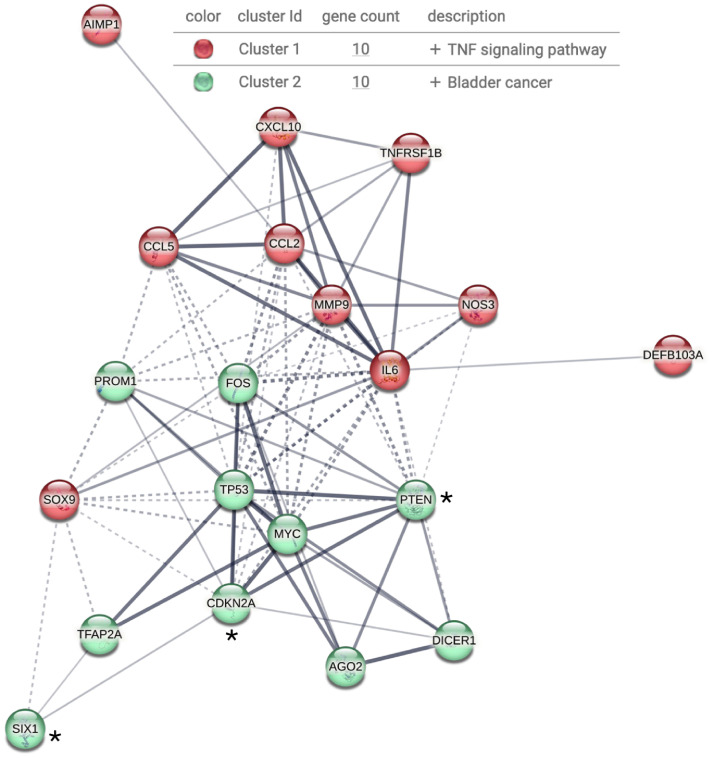
Interacting network of altered genes in lung tissues and cells derived from lung malignancies from PLWH. The list of genes from Table 1 was analyzed in STRING, Version 12.0. Two clusters were identified that, as expected, are related with cancer development and the TNF signaling pathway involving proinflammatory genes. As mentioned in the main text, *PTEN, *SIX1 and p16^INK4a^ (also known as *CDKN2A) are potential biomarkers of lung cancer prognosis.TP53 is synonym of p53.

The molecular interplay between HIV infection and lung cancer development involves a complex network of viral proteins, host signaling pathways, and epigenetic regulators (Fig. 3 and Table 1). Proteins such as Tat, Nef, and gp120 modulate oncogenic processes, including cell cycle dysregulation, apoptosis inhibition, epithelial-mesenchymal transition, angiogenesis, and immune evasion allowing virus persistence. Persistent viral reservoirs within lung tissue sustain local inflammatory and immunosuppressive microenvironments that stimulate cell transformation. Concurrently, HIV integration into host DNA and increased gene methylation contribute to the silencing of tumor suppressor genes. Also, extracellular vesicles carrying viral components maintain pro-tumorigenic signaling.

Future research should prioritize the construction of comprehensive interaction networks to enable the identification of molecular causal factors driving lung cancer progression in PLWH. Such integrative approaches may help to identify early prognostic biomarkers. At present, molecules such as PTEN, SIX1 and p16^INK4a^ have been described as prognostic biomarkers in human lung cancer, while Ago2 and DICER1 might be considered as molecules with potential prognostic application in this type of malignancy. From a therapeutic perspective, focusing on these biomarkers may provide a rationale for future studies to explore new strategies to combine antiretroviral drugs with targeted inhibitors of key effectors in oncogenic signaling pathways. In addition, public-health campaigns could be developed to emphasize the harmful effects of smoking, particularly among PLWH, to reduce predisposing factors for lung cancer.

## Authors' contributions

BRID, FNJ and RTE designed, collected data and wrote the manuscript; RHDL contributed to writing and prepared illustrations. All authors contributed to the article and approved the submitted version.

## Funding

This work was supported by the Secretaría de Ciencia, Humanidades, Tecnología e Innovación (SECIHTI), Mexico, project CBF2023-2024-2356.

## Data availability

The data that support the findings of this study are available from the corresponding author upon reasonable request.

## Conflicts of interest

The authors declare no conflicts of interest.
